# Effects of a novel curcumin derivative on insulin synthesis and secretion in streptozotocin-treated rat pancreatic islets *in vitro*

**DOI:** 10.1186/1749-8546-9-3

**Published:** 2014-01-14

**Authors:** Mohammed Talaat Abdel Aziz, Mohammed Farid El-Asmar, Ameen Mahmoud Rezq, Mohammed Abdel Aziz Wassef, Hanan Fouad, Nagwa Kamal Roshdy, Hanan Hosni Ahmed, Laila Ahmed Rashed, Dina Sabry, Fatma Mohammed Taha, Amira Hassouna

**Affiliations:** 1 Medical Biochemistry Department, Faculty of Medicine, Cairo University, POB 11562, Cairo, Egypt; 2 Medical Biochemistry Department, Faculty of Medicine, Ain Shams University, Cairo, Egypt

## Abstract

**Background:**

Hyperglycemia induces activation of the c-Jun N-terminal kinase (JNK) pathway, which suppresses insulin gene expression and reduces DNA binding of pancreatic and duodenal homeobox factor (PDX)-1. This study aims to investigate the effects of a novel curcumin derivative (NCD) on JNK signaling pathway on insulin synthesis and secretion in streptozotocin (STZ)-treated rat pancreatic islets *in vitro*.

**Methods:**

Isolated rat pancreatic islets were divided into five groups: untreated control group; group treated with NCD (10 μM); group exposed to STZ (5 mM); group treated with NCD (10 μM) and then exposed to STZ (5 mM); and group exposed to STZ (5 mM) and then treated with NCD (10 μM). The pancreatic islets from all groups were used for DNA fragmentation assays and quantitative assessments of the JNK, Pdx1, glucose transporter-2 (GLUT2), heme oxygenase (HO)-1, transcription factor 7-like 2 (TCF7L2), and glucagon-like peptide (GLP)-1 gene expression levels. The intracellular calcium, zinc, and the phosphorylated and total JNK protein levels were assessed. The insulin (secreted/total) and C-peptide levels were examined in islet culture medium.

**Results:**

NCD protected pancreatic islets against STZ-induced DNA damage, improved total insulin (*P* = 0.001), secreted insulin (*P* = 0.001), and C-peptide levels (*P* = 0.001), normalized mRNA expressions of insulin, Pdx1, and GLUT2 (*P* = 0.0001), and significantly elevated calcium and zinc levels (*P* = 0.0001). All effects were significant when islets were treated with NCD before STZ (*P* = 0.05). JNK gene overexpression and JNK protein levels induced by STZ were significantly inhibited after NCD treatment of islets ( *P* = 0.0001). NCD-treated islets showed significantly elevated gene expressions of HO-1, TCF7L2, and GLP-1 (*P* = 0.0001), and these upregulated gene expressions were more significantly elevated with NCD treatment before STZ than after STZ (*P* = 0.05).

**Conclusions:**

NCD improved insulin synthesis and secretion *in vitro* in isolated pancreatic islets treated with STZ through inhibition of the JNK pathway, up-regulation of the gene expressions of HO-1, TCF7L2, and GLP-1 and enhancing effects on calcium and zinc levels.

## Background

Pancreatic islet cell death is one cause of deficient insulin production in diabetes mellitus and prevention of this cell death is an important prophylactic measure in the control and management of hyperglycemia [1]. C-Jun N-terminal kinase (JNK/SAPK), a member of the mitogen-activated protein kinase family, can be rapidly activated by environmental stresses and inflammatory cytokines [2,3], and induce reactive oxygen species (ROS) generation in target cells [4,5].

The activation of the JNK pathway by ROS induces nucleocytoplasmic translocation of the transcription factor pancreatic and duodenal homeobox factor (PDX)-1, and decreases PDX-1 DNA binding activity, leading to pancreatic cell dysfunction [6]. PDX-1 (also known as IPF-1, IDX-1, and STF-1) is a homeodomain-containing transcription factor that is involved in the development and differentiation of the pancreas and duodenum [7-10]. This transcription factor was expressed before insulin during the ontogeny of the mouse pancreas, and restricted the β-cells and some α-cells in the adult pancreas [7,9,10]. In β-cells, PDX-1 binds to the A-element motif of the insulin gene and contributes to its β-cell-specific gene expression [7,9,10]. PDX-1 was also involved in glucokinase [6] and glucose transporter-2 (GLUT2) [11] gene expressions.

Kanitkar *et al.*[12,13] demonstrated that curcumin protected pancreatic islets against streptozotocin (STZ)-induced death or dysfunction. It also protected against cytokine-induced islet cell death or dysfunction by promoting the relocalization of NF-κB p65 into the cytoplasm, and prevents multiple low-dose STZ-induced diabetes in C57/BL6J mice [12,13].

Curcumin inhibited the JNK activation induced by carcinogens [14]. Curcumin was cytoprotective for pancreatic islet cells *via* inhibition of islet apoptosis, as it inhibited inflammatory cytokines and oxidative stress [15-17]. Curcumin induced heme oxygenase (HO)-1 synthesis, which enhanced cAMP synthesis to stimulate insulin release [18,19], and inhibited JNK, which was a signaling molecule linking inflammation to insulin resistance [20]. Curcumin significantly increased transcription factor 7-like 2 (TCF7L2) gene expression, which played a role in insulin release in pancreatic islets [21].

The systemic bioavailability of orally administered curcumin was relatively low in human. After oral administration (500 mg/kg), curcumin was present in plasma at levels near the detection limit (1.5 μM) [22]. Several water-soluble curcumin derivatives were prepared to achieve clinically efficient systemic bioavailability and a novel curcumin derivative (NCD) was developed through covalent modification of the curcumin molecule on sites remote from its natural functional groups.

This study aims to investigate the effect of a novel curcumin derivative (NCD) on JNK signaling pathway on insulin synthesis and secretion in streptozotocin (STZ)-treated rat pancreatic islets *in vitro*.

## Methods

### Synthesis of novel curcumin derivatives

The water-soluble NCD was developed through covalent modification of the curcumin molecule on sites remote from its natural functional groups. The NCD was presented free of charge to the participating researchers as a personal non-profit scientific participation in the present study. The novel derivative (WO/2011/100984) was registered as an international patent protected by the rights of “The Patent Cooperation Treaty” and is the personal property of its inventors, Rezq *et al.*[23].

Curcumin (Sigma Aldrich, USA), 1,7-bis (4-hydroxy-3-methoxyphenyl) -1,6-heptadiene-3,5-dione (I) (Sigma Aldrich, USA) was coupled to diazotized 4-aminobenzoic acid (Sigma Aldrich, USA). For synthesis of the novel compound 1,7-bis(5-carboxyphenylazo-4-hydroxy-3-methoxyphenyl)-1,6-heptadiene-3,5-dione (II), which in turn was utilized for synthesis of the novel curcumin-gelatin as a glutinous conjugate (III), through the use of 1-ethyl-3-(3-dimethylaminopropyl) carbodiimide hydrochloride (Sigma Aldrich, USA) (EDC). Both compounds (II) and (III) represent the novel curcumin derivative uder study.

Details could be found at the Additional file 1 (http://patentscope.wipo.int/search/en/detail.jsf%3Bjsessionid+90C05C7570FE02B89EBE9013FC25D794.wapp1?docId=WO2011100984&recNum=72&office=&queryString=&prevFilter=&sortOption=Pub+Date+Desc&maxRec=8021524).

Nitrous acid was generated by addition of a solution of 0.85 mEq of sodium nitrite (Sigma Aldrich, USA). to an excess of 1 N HCl with continuous stirring in an ice bath at 5°C. A solution of 0.85 mEq of 4-aminobenzoic acid in 1 N HCl (Sigma Aldrich, USA) chilled to 5°C was prepared with continuous stirring in an ice bath for 20 min, during which time the pH of 1.0 was never exceeded. The 4-aminobenzoic acid solution (Sigma Aldrich, USA) was then added slowly to the cold freshly prepared nitrous acid with continuous stirring in an ice bath at 5°C. Diazotized 4-aminobenzoic acid was added in a dropwise manner to an equivalent concentration (0.85 mEq) of curcumin (I) dissolved in ethanol (Sigma Aldrich, USA). /1 N NaOH (Sigma Aldrich, USA) at pH 11.0 with continuous stirring at 5°C. The solution was acidified with 1 N HCl to pH 2.0 at which point the derivative (II) was precipitated. The precipitate was centrifuged at 600 × *g*. (Beckman Coulter, Inc. USA) and redissolved in ethanol/1 N NaOH at pH 11.0. After repeating the acid-and-base cycle twice, the crude derivative (II) was chromatographed on a column of silica gel (Thermo Fisher Scientific Inc. USA). Reduced pressure and temperature evaporation of the elution solvent gave a derivative of about 98% purity, as checked by thin-layer chromatography.

The curcumin-gelatin conjugate (III) was synthesized in a medium of 1% NaCl/1,4-dioxane (Sigma Aldrich, USA) 1 N NaOH solution at pH 8–10, with continuous stirring at 5°C, by adding a pre-cooled (5°C) 0.1 M solution of 1-ethyl-3-(3-dimethylaminopropyl) carbodiimide hydrochloride, EDC, to the equivalent concentration of purified crystalline derivative (II) in the same medium with continuous stirring. A 1% gelatin solution (Sigma Aldrich, USA) in 0.5 N NaOH was added to the foregoing mixture at 5°C and pH 8–10 with continuous stirring for 1 h until the intermediate, azopseudourea had been completely conjugated to gelatin, as evidenced by complete disappearance of the original red color of the derivative (II) solution. Subsequently, the mixture was centrifuged at 600 × *g* , acidified to pH 5.1, salted out with solid NaCl (Sigma Aldrich, USA) or ammonium sulfate (Sigma Aldrich, USA), recentrifuged at 600 × *g*, redissolved, and dialyzed for 24 h at 5°C against 0.5 M sodium carbonate (Sigma Aldrich, USA) pH 8.2 until no color appeared in the dialysis solution. A final dialysis was performed against double-distilled water for 24 h at 5°C, after which the protein conjugate (III) was lyophilized.

### Reagents

STZ and collagenase were purchased from Sigma-Aldrich Corporation (St Louis, MO, USA). RPMI 1640 medium with HEPES, glucose, bicarbonate, and fetal calf serum was purchased from Invitrogen (Carlsbad, CA, USA).

### Experimental animals

The study was performed on adult female rats weighing 100–150 g obtained from an inbred colony (Curl: HEL1) at the Kasr Al-Aini Animal Experimental Unit, Faculty of Medicine, Cairo University. All animal care protocols were in accordance with and approved by the Institutional Animal Ethics Committee. The animals were kept in an environment with controlled temperature (25°C), humidity (45–75%), and photoperiod (12-h/12-h light/dark cycle). All animals had free access to chow and water.

### Isolation of pancreatic islets

Pancreatic islets were aseptically isolated from rat pancreases according to the optimized protocol described by Shewade *et al.*[24]. Aseptically excised rat pancreases were minced into three 1-mm pieces and digested with collagenase (Sigma Aldrich, USA) (1 mg/mL) for 10 min. The collagenase was then inactivated with two washes of RPMI 1640 containing 10% fetal calf serum (Sigma Aldrich, USA) and the samples were seeded into the same medium at one pancreas per flask. The primary cultures were incubated at 37°C under 5% CO_2_ for 48 h.

The pancreatic islets were divided into five experimental groups that each consisted of at least 150 islets. The first group was left untreated (untreated control). The second group was treated with NCD (10 μM) for 24 h (NCD control). The third group was exposed to STZ (5 mM) for 1 h at 37°C. The STZ solution was prepared in phosphate-buffered saline (Sigma Aldrich, USA). The fourth group was pretreated with NCD (10 μM) and then exposed to STZ (5 mM) for 1 h. The fifth group was exposed to STZ (5 mM) for 1 h and then treated with NCD (10 μM). The insulin (total/secreted), C-peptide, calcium, and zinc levels in islets were assessed after 1 h of NCD treatment, while the gene expression parameters were assessed after 4 h of NCD treatment.

### Estimation of insulin

For the total insulin content, pancreatic insulin was extracted according to Keong Tan *et al*. [25]. Thawed pancreas portion (0.2 g) was placed in a centrifuge tube containing 5.0 mL of ice-cold acid-alcohol solution. The mixture was homogenized for 3 min, followed by a 1-min sonication. The solution was left to stand at -20°C overnight and then centrifuged at 600 × *g* at 4°C for 15 min. The supernatant was transferred to a new centrifuge tube and stored at -20°C, while the pellet was subjected to another extraction. Before the insulin assay, the insulin extract was allowed to equilibrate to room temperature. Determination of the insulin content was performed by ELISA analytical kits (BioVendor GmbH, Heidelberg, Germany). The pancreatic insulin content was expressed as μg/mg wet tissue.

For the secreted insulin assay, 150 selected islets of roughly 150 μm in size from each experimental group were incubated in Krebs-Ringer buffer with HEPES (KRBH) containing 5.5 mM glucose at 37°C for 1 h, and the supernatants were collected.The islets were incubated in KRBH containing 16.5 mM glucose for 1 h, and the supernatants were collected to determine the insulin secretion responsiveness after stimulation with a high glucose concentration. All supernatants were stored at -80°C. The insulin concentrations were estimated by ELISA. The insulin levels in islets were assessed after 1 h of NCD treatment.

### Assessments of calcium and zinc

The calcium and zinc levels were assessed in the islet culture medium after 1 h of NCD treatment by colorimetric methods. The analytical kits were supplied by Quimica Clinica Aplicada SA (Amposta, Spain).

### Assessment of C-peptide

The C-peptide levels were assessed in the islet culture medium by an ELISA analytical kit (Monobind Inc. Lake Forest, Ca, USA).

### DNA fragmentation assay

One hundred and fifty pancreatic islets were collected and analyzed by agarose gel electrophoresis (Biometra, Göttingen, Germany) after protein and RNA digestion, as described previously [26].

### Gene expression protocol

After 4 h of NCD treatment, islets were separated from different buffers for measurements of the mRNA expression levels of JNK, insulin, Pdx1, GLUT2, HO-1, TCF7L2, and glucagon-like peptide (GLP)-1.

### RNA extraction

Total RNA was isolated from homogenized islets in the different groups by the RNeasy Purification Reagent (Qiagen, Valencia, CA, USA) according to the manufacturer’s protocol. The extracted RNA was quantified by spectrophotometry (JENWAY, USA) at 260 nm.

### Reverse transcription

The extracted RNA was reverse-transcribed into cDNA by a Reverse Transcription System Kit (Cat. no. A3500; Promega, Madison, WI, USA). The cDNA was generated from 5 μg of total RNA extracted with 1 μL (20 pmol) of antisense primer and 0.8 μL of superscript AMV reverse transcriptase for 60 min at 37°C.

### Real-time quantitative analyses

The relative abundances of the mRNA species were assessed by the SYBR® Green method and an ABI Prism 7500 Sequence Detector System (Applied Biosystems, Foster City, CA, USA). The PCR primers used were designed with Gene Runner Software (Hastings Software Inc., Hastings, NY, USA) from RNA sequences in GenBank (Table 1). All of the primer sets had a calculated annealing temperature of 60°C. Quantitative RT-PCR analyses were performed in duplicate in a 25-μL reaction volume consisting of 2× SYBR Green PCR Master Mix (Applied Biosystems, USA), 900 nM of each primer, and 2–3 μL of cDNA. The amplification conditions were 2 min at 50°C, 10 min at 95°C, and 40 cycles of denaturation at 95°C for 15 s and annealing/extension at 60°C for 10 min. Data from the real-time assays were calculated by Sequence Detection Software version 1.7 (PE Biosystems, Foster City, CA, USA). The relative expression levels of JNK, insulin, Pdx1, GLUT2, HO-1, TCF7L2, and GLP-1 were calculated by the comparative Ct method as stated by the manufacturer recommendations (Applied Biosystems, USA). All values were normalized to the expression of the β-actin gene and reported as the fold changes.

**Table 1:** Sequences of oligonucleotide primers used for real-time PCR

**Gene**	**Primer sequence**
Insulin	Forward primer: 5′- TCACACCTGGTGGAAGCTTC-3′
	Reverse primer: 5′- ACAATGCCACGCTTCTGC -3′
JNK	Forward primer: 5′- AAGCAGCAAGGCTACTCCTTCTCA-3′
	Reverse primer: 5′- ATCGAGACTGCTGTCTGTGTCTGA-3′
PDX-1	Forward primer: 5′-GGATGAAATCCACCAAAGCTC -3′
	Reverse primer: 5′- TTCCACTTCATGCGACGGT -3′
GLUT-2	Forward primer: 5′- CAAGATCACCGGACCTTGG -3′
	Reverse primer: 5′- ATTCCGCCTACTGCAAAGCT -3′
GLP-1	Forward primer: 5′- ACCTTCACCAGCGACGTAAG -3′
	Reverse primer: 5′- TCCTTTTACAAGCCAAGCGA – 3′
TCF7L2	Forward primer: 5′- CCGCCCGAACCTCTAACAAA - 3′
	Reverse primer: 5′ - TCAGTCTGTGACTTGGCGTC - 3′
HO-1	Forward primer: 5′- CTGTTGGCGACCGTGGCAGT – 3′
	Reverse primer: 5′- CTGGGCTCAGAACAGCCGCC – 3′
β-Actin	Forward primer: 5′-CCTTCCTGGGCATGGAGTCCT-3′
	Reverse primer: 5′- GGAGCAATGATCTTGATCTTC-3′

### Assessments of phosphorylated JNK and total JNK

An ELISA-based assay kit with a fluorogenic substrate was used to assess the phosphorylated and total JNK levels in islet cells in accordance with the manufacturer’s recommendations. The kit was supplied by R&D Systems (Minneapolis, MN, USA; Cat. no. KCB1205). The results were expressed as relative fluorescence units (RFUs) after subtracting the background fluorescence from the sample wells. Normalized results were determined by dividing the phospho-JNK fluorescence at 600 nm in each well by the total JNK fluorescence at 450 nm in each well. The normalized duplicate readings for each sample were averaged. The antibodies in the kit provide the same results by western blotting, as stated by the manufacturer.

### Statistical analysis

All data were presented as the mean ± standard derivation (SD). Statistical analyses were performed by one-way ANOVA followed by Tukey’s HSD test. Differences were considered statistically significant for values of *P* < 0.05. All data were analyzed by SPSS PC-software version 15.0 for Microsoft Windows (SPSS Inc., Chicago, IL, USA).

## Results

### The effects of NCD on STZ-induced DNA fragmentation

**Figure 1:**
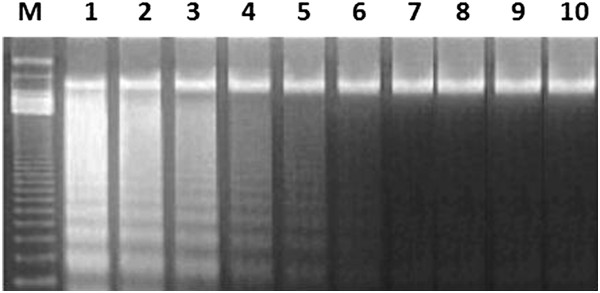
**Agarose gel electrophoresis of DNA isolated from cultured pancreatic β-cells.** Lane M: DNA markers (100 bp); lanes 1–3: STZ-treated group; lanes 4–5: STZ then NCD-treated group; lanes 6–7: NCD then STZ-treated group; lanes 8–9: control NCD-treated group; lane 10: control group.

The DNA fragmentation pattern was monitored in treated and untreated pancreatic islets by agarose gel electrophoresis. Necrotic strand breaks/streaking DNA was observed in islets treated by STZ, but not in islets pretreated with NCD prior to STZ exposure (Figure 1). These findings suggested that NCD had a cytoprotective effect against STZ damage. However, NCD treatment after STZ exposure did not protect the islets against DNA damage (Figure 1, lanes 4 and 5), suggesting that NCD would not have a prompt therapeutic effect. The control islet samples showed the presence of undamaged DNA, indicating that the mechanism of NCD-mediated protection against cell death may include prevention of DNA strand breaks.

### Effects of NCD on insulin secretion, C-peptide, and insulin gene expression

**Figure 2:**
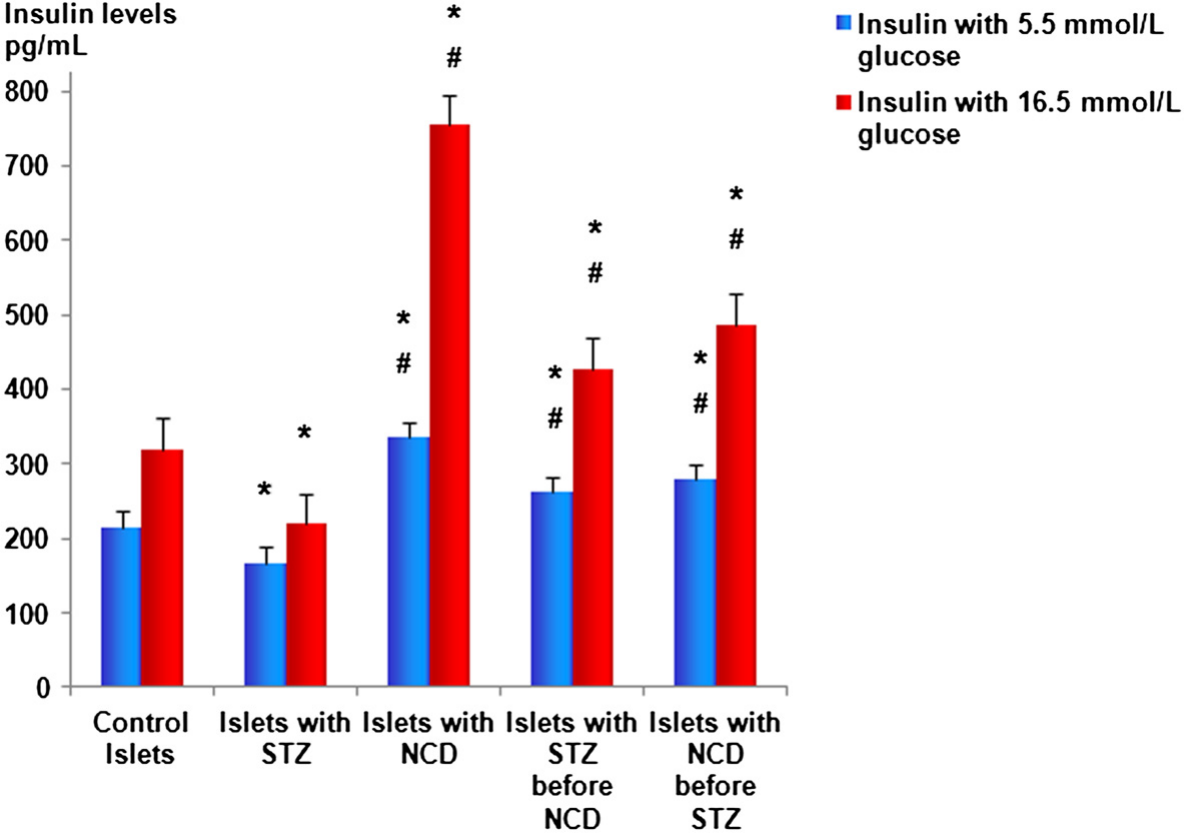
**Secreted insulin levels (pg/mL) after 1 h of NCD treatment.** Insulin secretion was measured at 5.5 and at 16.5 mM glucose. *Significant difference between all islet groups versus control islets; ^#^significant difference between all islet groups versus STZ-treated islets; ^##^significant difference between islets treated with NCD before STZ versus islets treated with NCD after STZ.

**Figure 3:**
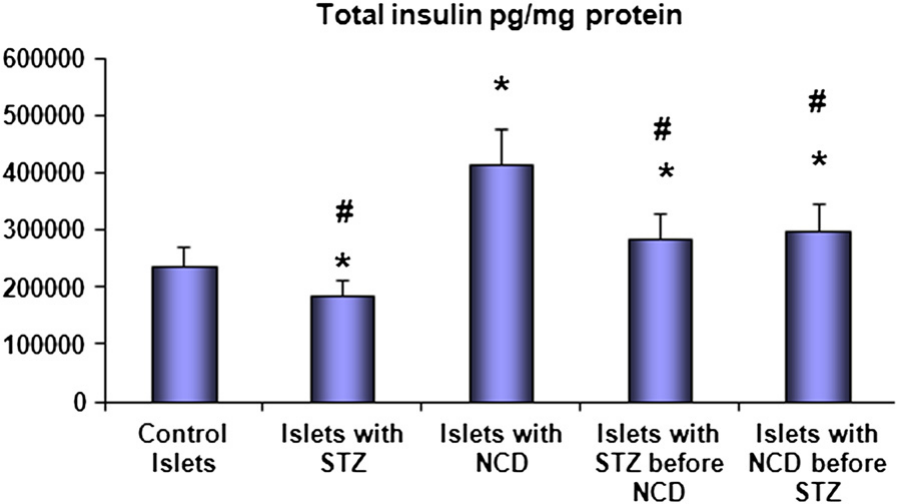
**Total insulin contents (pg/mg protein) after 1 h of NCD treatment.** Insulin secretion was measured at 16.5 mM glucose. *Significant difference between all islet groups versus control islets; ^#^significant difference between all islet groups versus NCD-treated group.

**Figure 4:**
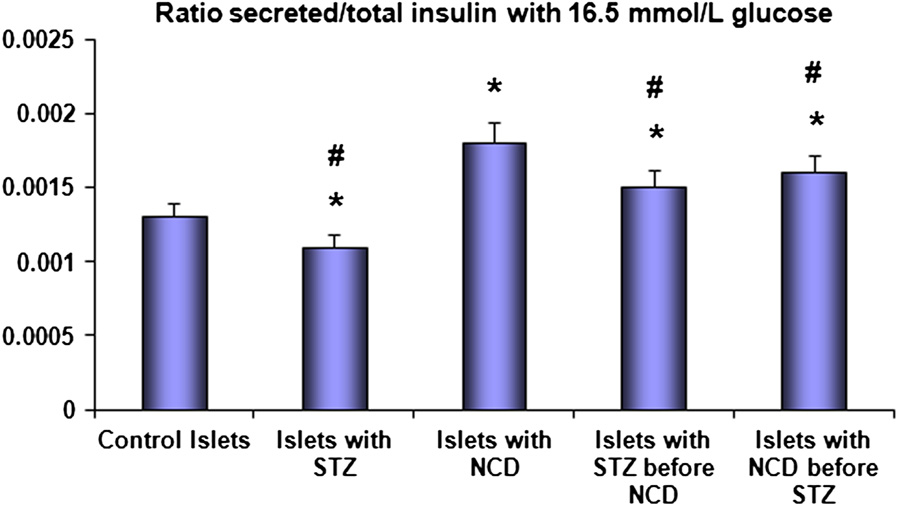
**Ratios between secreted insulin and total insulin after 1 h of NCD treatment.** Insulin secretion was measured at 16.5 mM glucose. *Significant difference between all islet groups versus control islets; ^#^significant difference between all islet groups versus NCD-treated group.

Insulin secretion was measured at the basal glucose concentration of 5.5 mM and the high glucose concentration of 16.5 mM (Figure 2), with the values expressed as pg/mL. Control islets treated with NCD showed the highest insulin secretion (335.3 ± 29.1 pg/mL for the basal glucose concentration and 754.15 ± 120.73 pg/mL for the high glucose concentration) among all the groups, and the differences were statistically significant (*P* = 0.001). Treatment with STZ caused significant decreases in insulin secretion to 178.03 ± 18.18 pg/mL and 220.15 ± 33.24 pg/mL for the basal and high glucose concentrations, respectively, compared with control islets (*P* = 0.001). The intracellular insulin levels showed similar patterns to the secreted insulin levels (Figure 3 and Figure 4).

**Figure 5:**
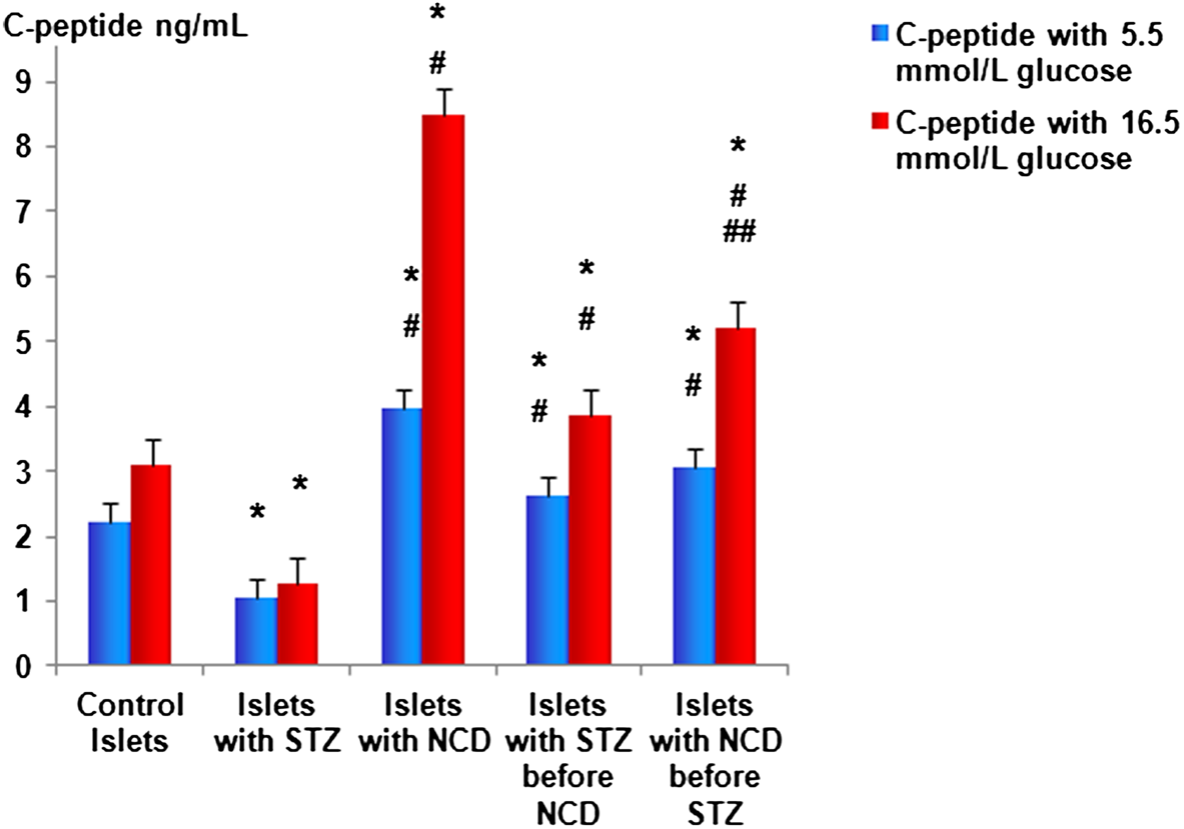
**C-peptide levels (ng/mL) after 1 h of NCD treatment.** C-peptide levels were measured at 5.5 and at 16.5 mM glucose. The results are presented as means ± SD. *Significant difference between all islet groups versus control islets; ^#^significant difference between all islet groups versus STZ-treated islets; ^##^significant difference between islets treated with NCD before STZ versus islets treated with NCD after STZ.

**Figure 6:**
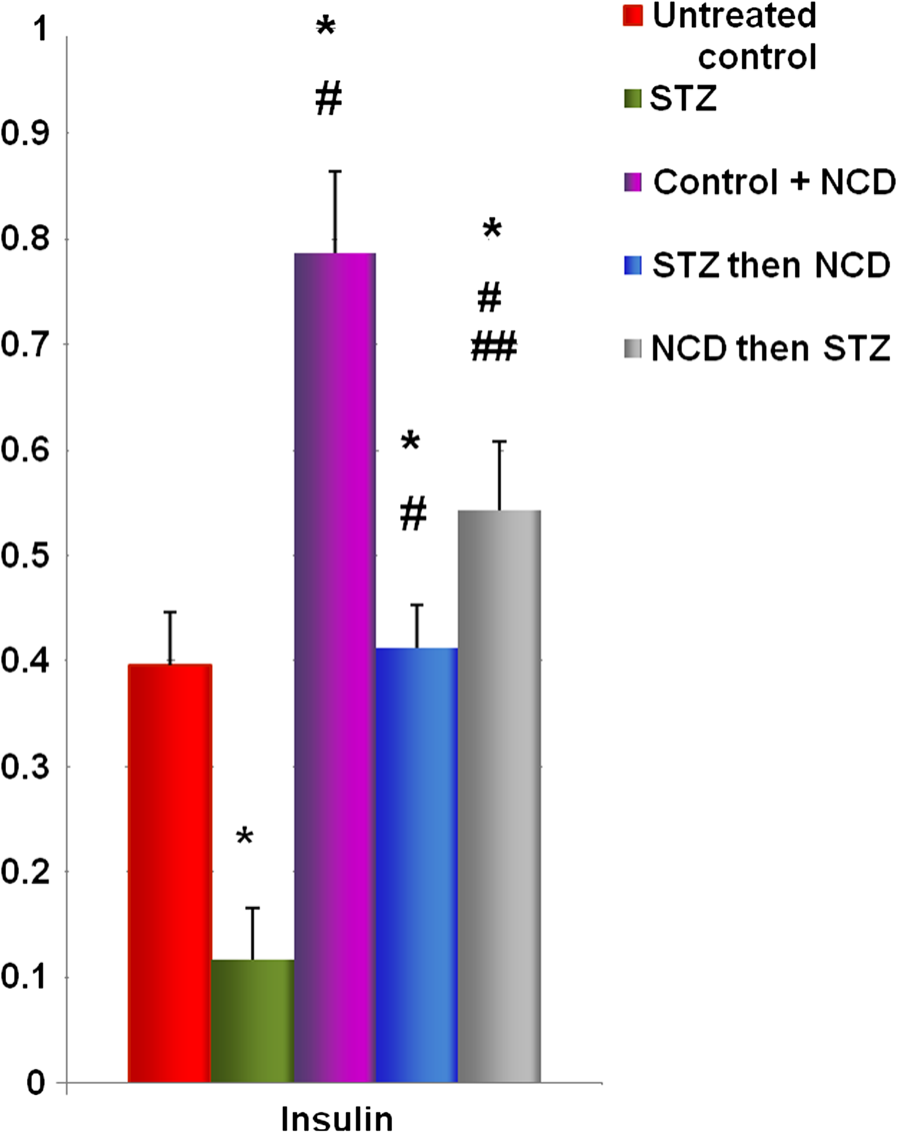
**Real-time PCR of insulin gene expression levels in Ct values relative to a housekeeping gene.** *Significant difference between all islet groups versus control islets; ^#^significant difference between all islet groups versus STZ-treated islets; ^##^significant difference between islets treated with NCD before STZ versus islets treated with NCD after STZ.

Pretreatment with NCD followed by STZ caused significant increases in insulin secretion to 279.9 + 39.05 pg/mL and 487.46 ± 99.89 pg/mL for the basal and high glucose concentrations, respectively, compared with control islets, corresponding to increases of 1.57- and 2.22-fold compared with STZ-treated islets (*P* = 0.001). For the STZ followed by NCD treatment group, the insulin secretion was significantly increased to 262.05 ± 0.02 pg/mL and 428.16 ± 47.58 pg/mL for the basal and high glucose concentrations, respectively, compared with the control group, corresponding to increases of 1.47- and 1.945-fold compared with STZ-treated islets (*P* = 0.05) (Figures 3 and 4). Assessments of the C-peptide levels (*P* = 0.001) as well as the insulin gene expression levels (*P* = 0.0001) demonstrated similar patterns to the insulin levels (Figure 5 and 6).

### Gene expressions of PDX1, GLUT2, and JNK

**Figure 7:**
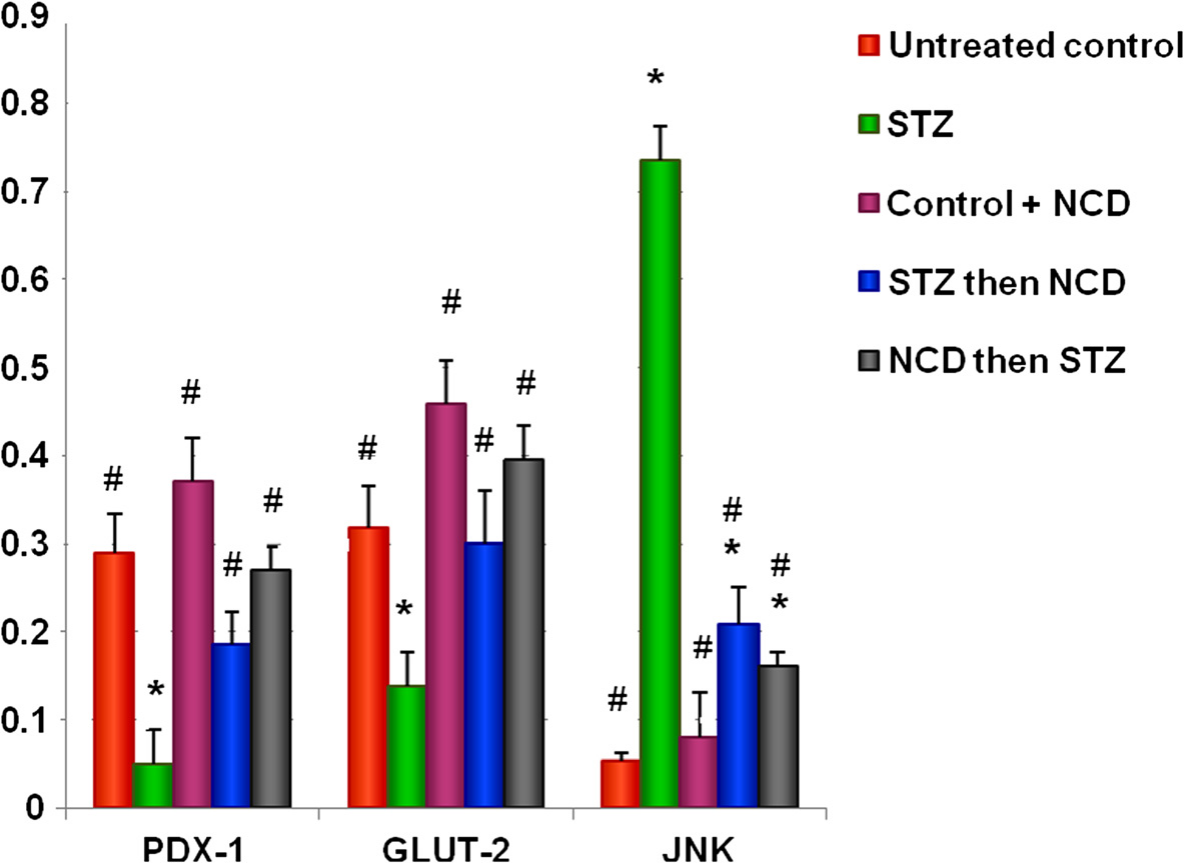
**Real-time PCR of PDX-1, GLUT2, and JNK gene expressions in Ct values relative to a housekeeping gene.** *Significant difference between all islet groups versus control islets; ^#^significant difference between all islet groups versus STZ-treated islets, ^##^significant difference between islets treated with NCD before STZ versus islets treated with NCD after STZ.

In STZ-treated islets, there were significant decreases in the gene expressions of PDX-1 and GLUT-2 and a significant elevation in the gene expression of JNK (*P* = 0.0001). Islets treated with NCD either before or after STZ treatment showed normalization of the gene expressions of PDX-1 and GLUT-2 (*P* = 0.0001), whereas the gene expression of JNK showed a significant decrease compared with STZ-treated islets, but was still higher than control islets (*P* = 0.001) (Figure 7).

### Gene expressions of HO-1, GLP-1, and TCF7L2

**Figure 8:**
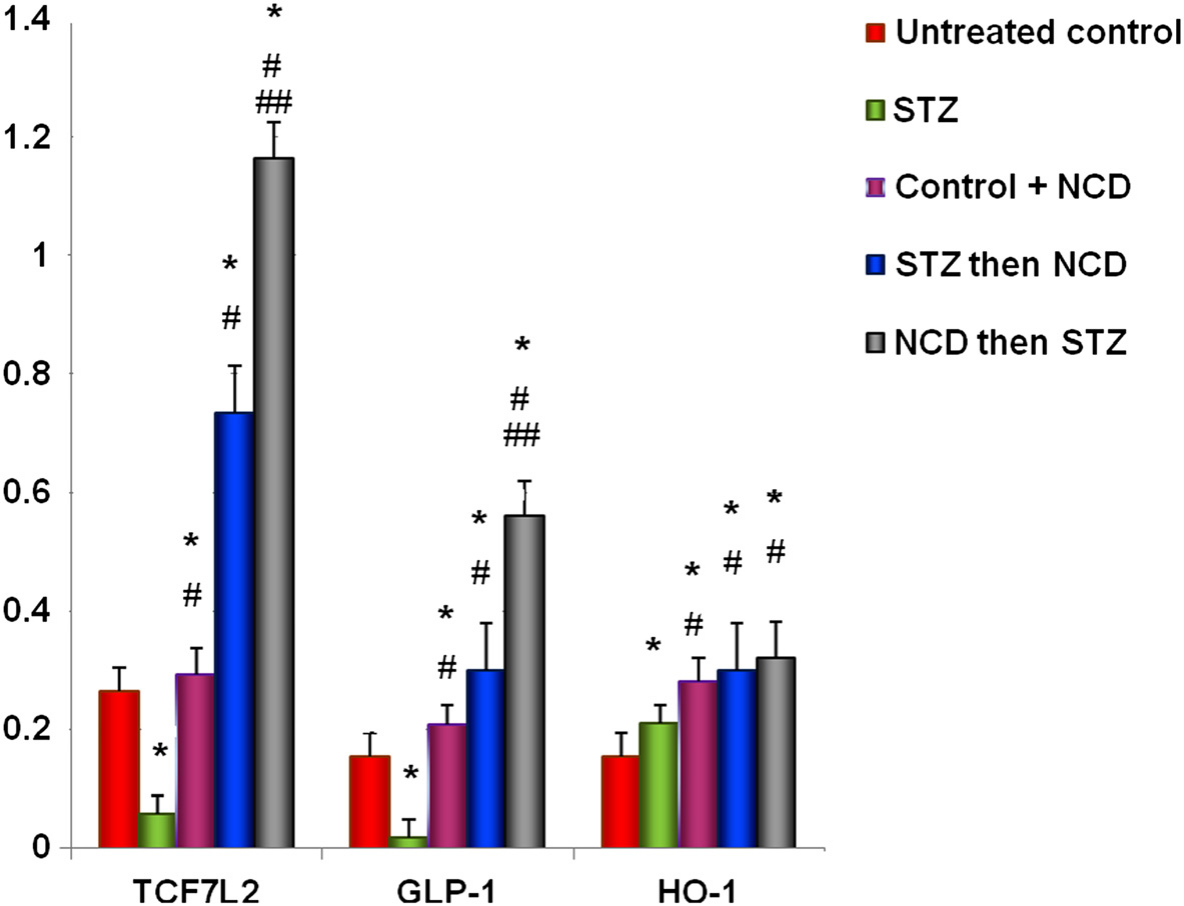
**Real-time PCR of TCF7L2, GLP-1, and HO-1 gene expressions in Ct values relative to a housekeeping gene.** *Significant difference between all islet groups versus control islets; ^#^significant difference between all islet groups versus STZ-treated islets; ^##^significant difference between islets treated with NCD before STZ versus islets treated with NCD after STZ.

Islets treated with STZ exhibited significant decreases in the gene expressions of GLP-1 and TCF7L2 (*P* = 0.0001) and a significant increase in the gene expression of HO-1 in comparison with control islets (*P* = 0.0001). All islet groups treated with NCD showed significant elevations in the gene expressions of HO-1 (*P* < 0.05), TCF7L2, and GLP-1, wherein the upregulation of the gene expressions was more significantly elevated when NCD was added to the islets before STZ exposure (*P* = 0.0001) than after STZ exposure (*P* = 0.05) (Figure 6). These findings indicate that NCD could have cytoprotective effects on islet cells (Figure 8).

### Phosphorylated and total JNK

**Figure 9:**
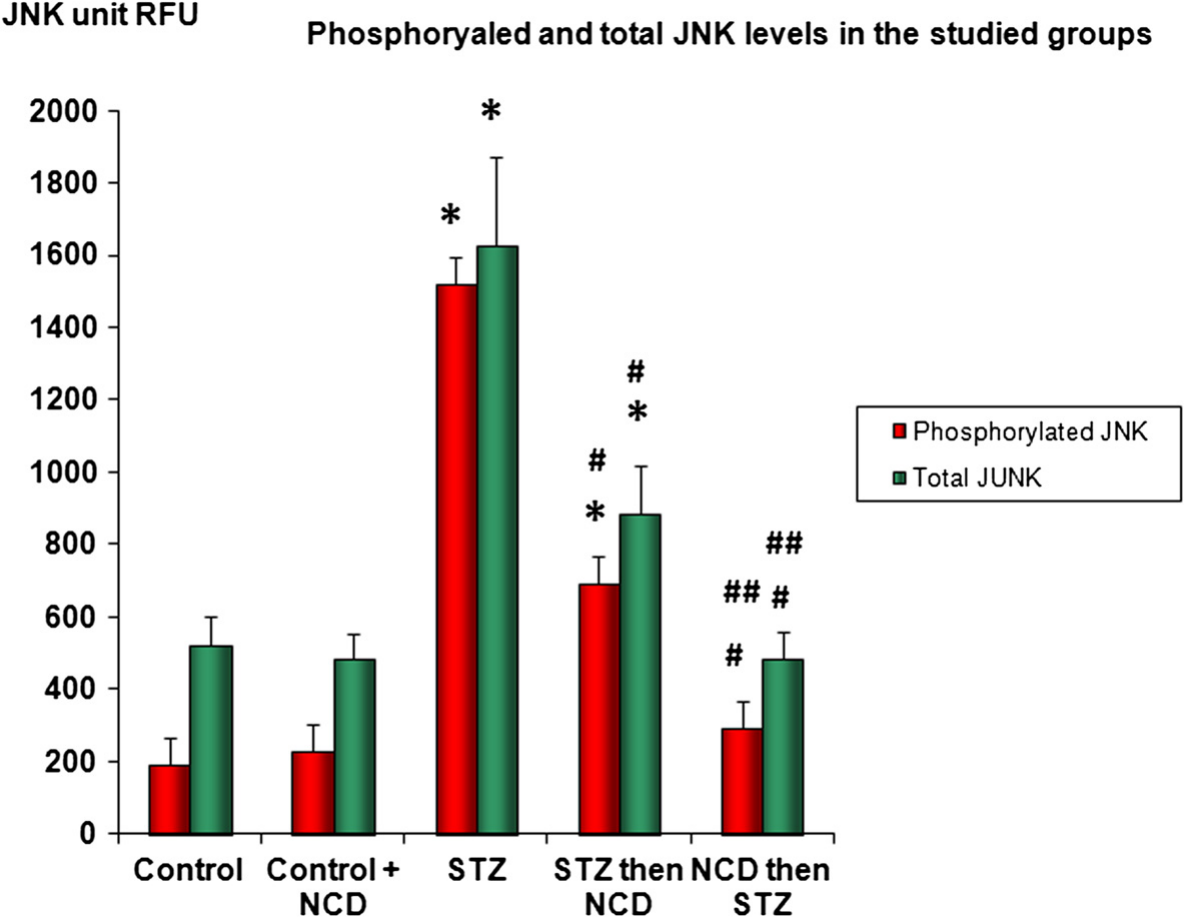
**Phospho-JNK and total JNK levels expressed as relative fluorescence units (RFUs).** *Significant difference between all islet groups versus control islets; ^#^significant difference between all islet groups versus STZ-treated islets; ^##^significant difference between islets treated with NCD before STZ versus islets treated with NCD after STZ.

**Figure 10:**
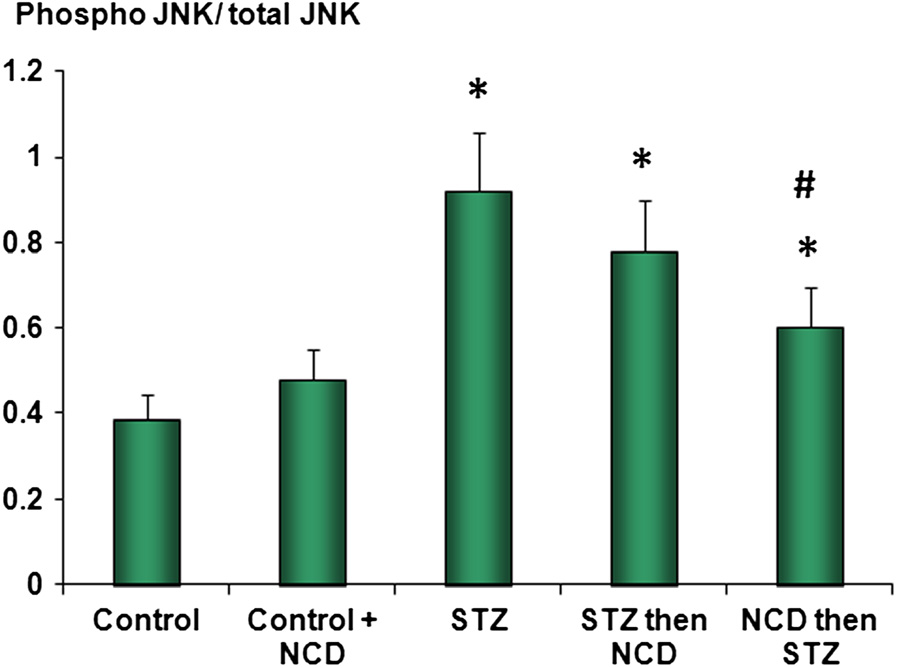
**Ratios of phospho-JNK/total JNK expressed as normalized RFUs determined by dividing the phospho-JNK fluorescence at 600 nm by the total JNK fluorescence at 450 nm in each well.** *Significant difference between all islet groups versus control islets; ^#^significant difference between all islet groups versus STZ-treated islets; ^##^significant difference between islets treated with NCD before STZ versus islets treated with NCD after STZ.

The results for the JNK protein assessments by ELISA were similar to those of the gene expression analyses, wherein both phospho-JNK and total JNK were significantly elevated in the STZ-treated islet group (*P* = 0.0001). Administration of NCD significantly decreased phospho-JNK and total JNK (*P* = 0.0001), with more superior effects when NCD was added to the islets before STZ exposure (*P* = 0.05) than after STZ exposure. With regard to the phospho-JNK/total JNK ratio, exposure of islets to NCD significantly decreased the ratio, but its levels were still higher than in control islets. A more superior effect was observed when NCD was added to islets before STZ exposure (*P* = 0.05) (Figures 9 and 10).

### Calcium and zinc levels

**Figure 11:**
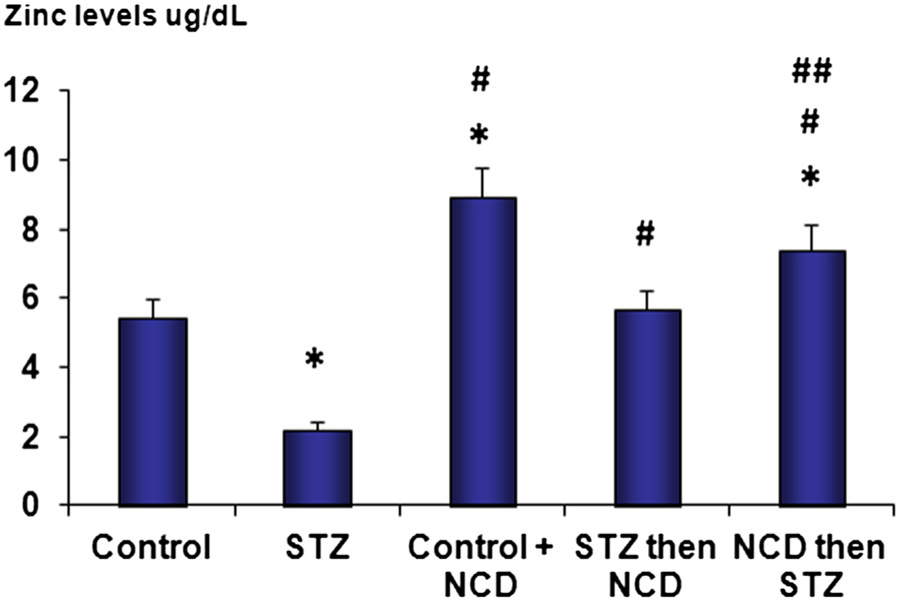
**Zinc levels (μg/dL) in islet groups.** *Significant difference between all islet groups versus control islets; ^#^significant difference between all islet groups versus STZ-treated islets; ^##^significant difference between islets treated with NCD before STZ versus islets treated with NCD after STZ.

**Figure 12:**
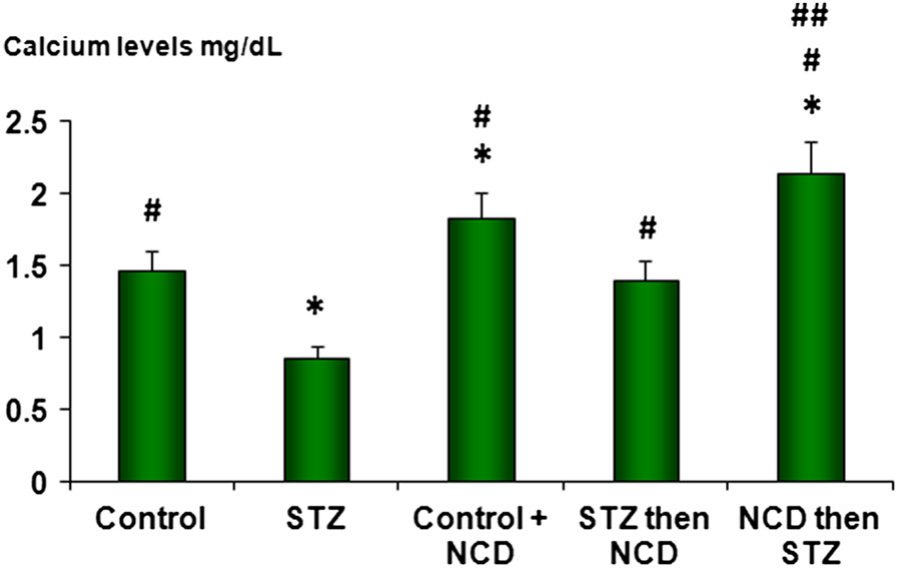
**Calcium levels (mg/dL) levels in islet groups.** *Significant difference between all islet groups versus control islets; ^#^significant difference between all islet groups versus STZ-treated islets; ^##^significant difference between islets treated with NCD before STZ versus islets treated with NCD after STZ.

In the STZ-treated islet group, significant decreases in the zinc and calcium levels were observed compared with the control group (*P* = 0.0001). NCD-treated islets and NCD-pretreated islets before STZ exposure showed significant elevations in the zinc and calcium levels in comparison with the control group (*P* = 0.0001). The effects of NCD pretreatment before STZ on the calcium and zinc levels (at 16.5 mM glucose) were significantly superior to those of NCD treatment after STZ exposure (*P* = 0.05) (Figures 11 and 12).

## Discussion

In the present study, the DNA fragmentation patterns in STZ-treated and untreated pancreatic islets were studied. STZ caused necrotic strand breaks of DNA, which were not observed in DNA isolated from islets pretreated with NCD prior to STZ exposure, suggesting that NCD has cytoprotective effects against STZ damage. However, partial DNA damage was detected in islets treated with NCD after STZ exposure, indicating that NCD would not have prompt therapeutic effects [27-29]. Chanpoo *et al.*[30] reported that curcumin treatment induced islet cell neogenesis and regeneration after 12 weeks in a diabetic mouse model.

NCD treatment either before or after STZ exposure increased insulin secretion, compared with STZ-treated islets. We previously demonstrated that there were significant elevations in insulin secretion by islets incubated for 1 and 4 h with different concentrations of curcumin, compared with control islets *in vitro*[18]. Intracellular insulin followed a similar pattern to secreted insulin. NCD supplementation to diabetic rats significantly lowered the plasma glucose by 27.5% and increased the plasma insulin by 66.67%, compared with control rats *in vivo*[19].

Kanitkar *et al.*[12] demonstrated the efficacy of curcumin in protecting pancreatic islets against STZ-induced death or dysfunction by retarding the generation of islet ROS along with inhibition of poly [ADP-ribose] polymerase-1 activation and preventing decreases in the free radical-scavenging enzymes such as Cu/Zn superoxide dismutase. In 2008, Kanitkar *et al.*[13] revealed that curcumin protected pancreatic islets against cytokine-induced death or dysfunction *in vitro* and prevented STZ-induced diabetes *in vivo*. Kanitkar and Bhonde [31] showed that inclusion of curcumin in islet cryopreservation medium enhanced islet viability after thawing and maintained islet functionality in culture.

There was a significant increase in JNK gene expression in STZ-treated islets compared with control islets. Treatment with NCD either before or after STZ exposure significantly decreased JNK gene expression. Chen and Tan [14] demonstrated that curcumin blocks JNK activation in a dose-dependent manner. JNKs were activated by phosphorylation in response to cellular stress and inflammatory cytokines [32,33]. T cell receptor signals were efficient for the induction of JNK gene expression, while JNK phosphorylation also required CD28-mediated costimulatory signals [34,35]. Both of these mechanisms were functional in type I diabetes during β-cell-induced damage.

Kaneto *et al.*[36] found that JNK overexpression suppressed insulin gene expression without affecting the c-Jun expression levels. The suppression of insulin gene expression by JNK overexpression was accompanied by decreased expression of PDX-1, which in turn caused downregulation of β-cell genes, such as insulin, GLUT2, and glucokinase [36,37]. These data coincided with our results, since the gene expressions of insulin, GLUT2, and PDX1 were significantly reduced in STZ-treated islets. There were significantly higher expression levels of insulin, GLUT2, and PDX1 in all NCD-treated islet groups, wherein insulin gene expression was significantly higher in islets pretreated with NCD and then treated with STZ compared with islets pretreated with STZ and then treated with NCD.

Kawamori *et al.*[38] investigated the possible effects of oxidative stress on the intracellular localization of the PDX-1 protein. They found that oxidative stress induces nucleocytoplasmic translocation of PDX-1 through activation of the JNK pathway [39,40]. The oxidative stress-induced nucleocytoplasmic translocation of PDX-1 may play a crucial role in the suppression of insulin gene expression and biosynthesis under diabetic conditions.

In the present study, the TCF7L2 and GLP-1 gene expressions were significantly decreased in STZ-treated islet cells. Treatment with NCD in control islets, and before or after STZ exposure significantly increased TCF7L2 and GLP-1 expressions. These findings were consistent with the results reported by Khalooghi *et al.*[21], who described that treatment of a pancreatic cell line with curcumin significantly upregulated TCF7L2 gene expression by 3.24-fold. Shu *et al.*[41] observed that TCF7L2 depletion with an siRNA resulted in a 5.1-fold increase in β-cell apoptosis, 2.2-fold decrease in β-cell proliferation, and 2.6-fold decrease in glucose-stimulated insulin secretion in human islets. In contrast, overexpression of TCF7L2 protected the islets from glucose and cytokine-induced apoptosis and impaired function [41]. TCF7L2 is implicated in glucose homeostasis through its regulation of expression of the proglucagon gene, which encodes GLP-1 that is directly involved in insulin release [42-45].

GLP-1 is a neuropeptide that binds to specific G-protein receptors, thereby activating adenylate cyclase and controlling a certain type of calcium channels called voltage-dependent calcium channels [46]. Regulation of these voltage-dependent calcium channels by GLP-1 could explain the elevation calcium levels in islet cells treated with NCD.

In this study, NCD increased the zinc levels in pancreatic islets whether the treatment was performed before or after STZ exposure. Elevation of zinc levels augments insulin synthesis and release. Malhotra *et al.*[47] and Kalpana and Menon [48] demonstrated that curcumin significantly enhanced zinc levels *via* specific signaling pathways. Moreover, Li [49] stated that Zn^2+^ was essential for the correct processing, storage, secretion, and action of insulin in pancreatic β-cells. Li’s study [49] indicated that secreted Zn^2+^ has autocrine and paracrine signaling effects on neighboring β-cells. Changes in Zn^2+^ levels in the pancreas have been found to be associated with diabetes.

Istyastono [50] stated that curcumin was an efficient of dipeptidyl peptidase (DPP) inhibitor -which inactivated GLP-1. Therefore, curcumin prevents the inactivation of GLP-1, which could enhance insulin secretion [51].

Moreover, Chuengsamarn *et al*. [52] showed that 9 months of treatment with curcumin led to higher homeostatic measurement assessment (HOMA)-β and a lower level of HOMA insulin resistance. Furthermore, curcumin induced electrical activity in pancreatic β-cells by activating the volume-regulated anion channel *f*, was accompanied by enhanced insulin release [53-55].

## Conclusions

NCD improved insulin synthesis and secretion *in vitro* in isolated pancreatic islets treated with STZ through inhibition of the JNK pathway, upregulation of the gene expressions of HO-1, TCF7L2, and GLP-1 and enhancing effects on calcium and zinc levels.

## Abbreviations

NCD: Novel curcumin derivative; STZ: Streptozotocin; JNK: c-Jun N-terminal kinase; PDX-1: Pancreatic and duodenal homeobox factor-1; GLUT2: Glucose transporter-2; HO-1: Heme oxygenase-1; TCF7L2: Transcription factor 7-like 2; GLP-1: Glucagon-like peptide 1; DPP-4: Dipeptidyl peptidase-4.

## Competing interests

The authors declare that they have no competing interests.

## Authors’ contributions

AAMT, EAMF, RAM, WMA, and FH designed the study. RNK, AHH, RL, SD, TFM, and HA performed the experiments. AAMT, EAMF, RAM, WMA, FH, RNK, AHH, RL, SD, TFM, and HA analyzed and interpreted the data. RNK, AHH, RL, SD, TFM, and HA wrote the manuscript. All authors read and approved the final version of the manuscript.

## Supplementary Material

Additional file 1 GELATIN, A CURCUMIN DRUG CARRIER SYSTEM. Click here for file
