# Supplementary material for: Effects of a novel curcumin derivative on insulin synthesis and secretion in streptozotocin-treated rat pancreatic islets in vitro
Source: Chin Med. 2014 Jan 14;9:3. doi: 10.1186/1749-8546-9-3 (PMC3896850; doi:10.1186/1749-8546-9-3)
Supplement: Additional file 1 — GELATIN, A CURCUMIN DRUG CARRIER SYSTEM. [file 1749-8546-9-3-S1.docx]

Islet merged Supplementary File

<http://patentscope.wipo.int/search/en/detail.jsf%3Bjsessionid+90C05C7570FE02B89EBE9013FC25D794.wapp1?docId=WO2011100984&recNum=72&office=&queryString=&prevFilter=&sortOption=Pub+Date+Desc&maxRec=8021524>

| [[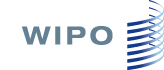](http://www.wipo.int/)](http://www.wipo.int) | \| Top of Form  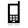[Mobile \|](http://patentscope.wipo.int/search/mobile;jsessionid=8633ED619BCF398226A5E614CF101D5A.wapp1) [Deutsch \|](http://patentscope.wipo.int/search/de/detail.jsf;jsessionid+90C05C7570FE02B89EBE9013FC25D794.wapp1;jsessionid=8633ED619BCF398226A5E614CF101D5A.wapp1?docId=WO2011100984&recNum=72&office=&queryString=&prevFilter=&sortOption=Pub+Date+Desc&maxRec=8021524) [Español \|](http://patentscope.wipo.int/search/es/detail.jsf;jsessionid+90C05C7570FE02B89EBE9013FC25D794.wapp1;jsessionid=8633ED619BCF398226A5E614CF101D5A.wapp1?docId=WO2011100984&recNum=72&office=&queryString=&prevFilter=&sortOption=Pub+Date+Desc&maxRec=8021524) [Français \|](http://patentscope.wipo.int/search/fr/detail.jsf;jsessionid+90C05C7570FE02B89EBE9013FC25D794.wapp1;jsessionid=8633ED619BCF398226A5E614CF101D5A.wapp1?docId=WO2011100984&recNum=72&office=&queryString=&prevFilter=&sortOption=Pub+Date+Desc&maxRec=8021524) [日本語 \|](http://patentscope.wipo.int/search/ja/detail.jsf;jsessionid+90C05C7570FE02B89EBE9013FC25D794.wapp1;jsessionid=8633ED619BCF398226A5E614CF101D5A.wapp1?docId=WO2011100984&recNum=72&office=&queryString=&prevFilter=&sortOption=Pub+Date+Desc&maxRec=8021524) [한국어 \|](http://patentscope.wipo.int/search/ko/detail.jsf;jsessionid+90C05C7570FE02B89EBE9013FC25D794.wapp1;jsessionid=8633ED619BCF398226A5E614CF101D5A.wapp1?docId=WO2011100984&recNum=72&office=&queryString=&prevFilter=&sortOption=Pub+Date+Desc&maxRec=8021524) [Português \|](http://patentscope.wipo.int/search/pt/detail.jsf;jsessionid+90C05C7570FE02B89EBE9013FC25D794.wapp1;jsessionid=8633ED619BCF398226A5E614CF101D5A.wapp1?docId=WO2011100984&recNum=72&office=&queryString=&prevFilter=&sortOption=Pub+Date+Desc&maxRec=8021524) [Русский \|](http://patentscope.wipo.int/search/ru/detail.jsf;jsessionid+90C05C7570FE02B89EBE9013FC25D794.wapp1;jsessionid=8633ED619BCF398226A5E614CF101D5A.wapp1?docId=WO2011100984&recNum=72&office=&queryString=&prevFilter=&sortOption=Pub+Date+Desc&maxRec=8021524) [中文 \|](http://patentscope.wipo.int/search/zh/detail.jsf;jsessionid+90C05C7570FE02B89EBE9013FC25D794.wapp1;jsessionid=8633ED619BCF398226A5E614CF101D5A.wapp1?docId=WO2011100984&recNum=72&office=&queryString=&prevFilter=&sortOption=Pub+Date+Desc&maxRec=8021524)  Bottom of Form \| \| --- \| \| **PATENTSCOPE** \| \| Search International and National Patent Collections \| |
| --- | --- | --- | --- | --- |
| 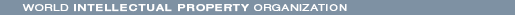 | |

Top of Form

| Search | 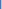 | Browse | 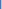 | Translate | 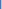 | Options | 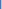 | News | 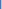 | Login | 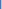 | Help |  |
| --- | --- | --- | --- | --- | --- | --- | --- | --- | --- | --- | --- | --- | --- |

Bottom of Form

- [Home](http://www.wipo.int/portal/index.html.en)
- [IP Services](http://www.wipo.int/services/en/)
- [PATENTSCOPE](http://www.wipo.int/patentscope/en/)

Top of Form

| **[⇦](http://patentscope.wipo.int/search/en/detail.jsf;jsessionid=8633ED619BCF398226A5E614CF101D5A.wapp1?recNum=71&queryString=&office=&sortOption=Pub+Date+Desc&prevFilter=&maxRec=8021524&navig=previous)** | **[⇧](http://patentscope.wipo.int/search/en/result.jsf;jsessionid=8633ED619BCF398226A5E614CF101D5A.wapp1?query=&office=&prevFilter=&sortOption=Pub+Date+Desc&maxRec=8021524)** | **[⇨](http://patentscope.wipo.int/search/en/detail.jsf;jsessionid=8633ED619BCF398226A5E614CF101D5A.wapp1?recNum=73&maxRec=8021524&office=&prevFilter=&sortOption=Pub+Date+Desc&queryString=&navig=next)** | [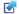](http://patentscope.wipo.int/search/en/detail.jsf;jsessionid=8633ED619BCF398226A5E614CF101D5A.wapp1?noMenu=false&docId=WO2011100984&recNum=73&maxRec=8021524&office=&prevFilter=&sortOption=Pub+Date+Desc&navig=existing&queryString=) |
| --- | --- | --- | --- |

Bottom of Form

**72. (WO2011100984) GELATIN, A CURCUMIN DRUG CARRIER SYSTEM**

Top of Form

| \|  \| \|  \| \| [PCT Biblio. Data](http://patentscope.wipo.int/search/en/detail.jsf;jsessionid=8633ED619BCF398226A5E614CF101D5A.wapp1?docId=WO2011100984&recNum=72&maxRec=8021524&office=&prevFilter=&sortOption=Pub+Date+Desc&queryString=&tab=PCT+Biblio) \| \| --- \| \|  \| \| --- \| --- \| --- \| --- \| \|  \| \|  \| \| [Description](http://patentscope.wipo.int/search/en/detail.jsf;jsessionid=8633ED619BCF398226A5E614CF101D5A.wapp1?docId=WO2011100984&recNum=72&maxRec=8021524&office=&prevFilter=&sortOption=Pub+Date+Desc&queryString=&tab=PCTDescription) \| \| --- \| \|  \| \| --- \| --- \| --- \| --- \| \|  \| \|  \| \| [Claims](http://patentscope.wipo.int/search/en/detail.jsf;jsessionid=8633ED619BCF398226A5E614CF101D5A.wapp1?docId=WO2011100984&recNum=72&tab=PCTClaims&maxRec=8021524&office=&prevFilter=&sortOption=Pub+Date+Desc&queryString=) \| \| --- \| \|  \| \| --- \| --- \| --- \| --- \| \|  \| \|  \| \| National Phase \| \| --- \| \|  \| \| --- \| --- \| --- \| --- \| \|  \| \|  \| \| [Notices](http://patentscope.wipo.int/search/en/detail.jsf;jsessionid=8633ED619BCF398226A5E614CF101D5A.wapp1?docId=WO2011100984&recNum=72&tab=Notices&maxRec=8021524&office=&prevFilter=&sortOption=Pub+Date+Desc&queryString=) \| \| --- \| \|  \| \| --- \| --- \| --- \| --- \| \|  \| \|  \| \| [Drawings](http://patentscope.wipo.int/search/en/detail.jsf;jsessionid=8633ED619BCF398226A5E614CF101D5A.wapp1?docId=WO2011100984&recNum=72&tab=Drawings&maxRec=8021524&office=&prevFilter=&sortOption=Pub+Date+Desc&queryString=) \| \| --- \| \|  \| \| --- \| --- \| --- \| --- \| \|  \| \|  \| \| [Documents](http://patentscope.wipo.int/search/en/detail.jsf;jsessionid=8633ED619BCF398226A5E614CF101D5A.wapp1?docId=WO2011100984&recNum=72&tab=PCTDocuments&maxRec=8021524&office=&prevFilter=&sortOption=Pub+Date+Desc&queryString=) \| \| --- \| \|  \| \| --- \| --- \| --- \| --- \| \|  \| \| --- \| --- \| --- \| --- \| --- \| --- \| --- \| --- \| --- \| --- \| --- \| --- \| --- \| --- \| --- \| --- \| --- \| --- \| --- \| --- \| --- \| --- \| --- \| --- \| --- \| --- \| --- \| --- \| --- \| --- \| --- \| --- \| --- \| --- \| --- \| --- \| --- \| --- \| --- \| --- \| --- \| --- \| --- \| |
| --- | --- | --- | --- | --- | --- | --- | --- | --- | --- | --- | --- | --- | --- | --- | --- | --- | --- | --- | --- | --- | --- | --- | --- | --- | --- | --- | --- | --- | --- | --- | --- | --- | --- | --- | --- | --- | --- | --- | --- | --- | --- | --- | --- |
| \| \| **Latest bibliographic data on file with the International Bureau**   - [PermaLink[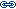](http://patentscope.wipo.int/search/en/WO2011100984)](http://patentscope.wipo.int/search/en/WO2011100984)    - [PermaLink](http://patentscope.wipo.int/search/en/WO2011100984)   - [Bookmark](javascript:bookmark('http://patentscope.wipo.int/search/en/WO2011100984)','GELATIN,%20A%20CURCUMIN%20DRUG%20CARRIER%20SYSTEM');) \| \| \| --- \| --- \| \|  \| \| \|  \|  \| \| \| \| **Pub. No.:** \|  \| WO/2011/100984 \|  \| **International Application No.:** \|  \| PCT/EG2010/000008 \| \| --- \| --- \| --- \| --- \| --- \| --- \| --- \| \| **Publication Date:** \| 25.08.2011 \| **International Filing Date:** \| 17.02.2010 \| \| \|  \| \| **IPC:** \| \| ***A61K 47/48*** (2006.01), ***A61P 15/10*** (2006.01), ***A61P 3/10*** (2006.01) \| 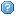 \| \| --- \| --- \| \|  \| \| **Applicants:** \| **REZQ, El-Sayed Ameen Mahmoud** [EG/SA]; (SA). **MANSOUR, Mohammad Talaat Abdel-Aziz** [EG/EG]; (EG). **AL-MALKI, Abdulrahman Labeed** [SA/SA]; (SA) \|  \| \| **Inventors:** \| **REZQ, El-Sayed Ameen Mahmoud**; (SA). **MANSOUR, Mohammad Talaat Abdel-Aziz**; (EG). **AL-MALKI, Abdulrahman Labeed**; (SA) \|  \| \| **Priority Data:** \| \|  \| \| --- \| \|  \| \| **Title** \| **(EN)** GELATIN, A CURCUMIN DRUG CARRIER SYSTEM **(FR)** GÉLATINE EN TANT QUE SYSTÈME DE VÉHICULE DE MÉDICAMENT DE CURCUMINE \|  \| \| **Abstract:** \| [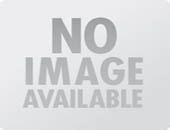](http://patentscope.wipo.int/search/docservice_fpimage/WOEG2010000008@@@false@@@en;jsessionid=8633ED619BCF398226A5E614CF101D5A.wapp1)  **(EN)**Curcumin is a water insoluble component of turmeric, the yellow spice from the rhizome of the herb *Curcuma longa* L. (Zingiberaceae), that is widely used as a food flavoring and coloring agent. Curcumin has a long history of medicinal use for a wide variety of medical conditions. The World Health Organization approved its use as non-toxic food additive and a potentially safe drug. Oral administration is well tolerated, but bioavailability is very low. Less than 1% of the curcumin makes its way to the bloodstream, where the liver rapidly destroys most of it. A suitable protein carrier system covalently linked to curcumin with conserved natural curcumin functional chemical groups, of free water solubility, easy digestibility, free intestinal absorption, long serum half-life should help getting the utmost possible beneficial effects of this historical, albeit promising treatment for several acute and chronic illnesses. Additionally, the protein-curcumun conjugate could be used as health food additive and a natural food colorant. Accordingly, based on the successful application of our previous "Patent Pending PCT/EG2008/000044" filed on 20/11/2008 under "Long Acting Conserved Natural Functional Groups Curcumin", the need for the present development looked urgent through the use of several other protein carrier systems for curcumin. The successful clinical application of a globular carrier protein system for curcumin has been internationally published in the Journal of International Society for Sexual Medicine and is available online since Oct. 19 2009 12:02PM, under "Novel Water-soluble Curcumin Derivative Mediating Erectile Signaling". **(FR)**La curcumine est un composant insoluble dans l'eau du curcuma, l'épice jaune provenant du rhizome de la plante *Curcuma longa* L. (Zingiberaceae), qui est largement utilisée en tant qu'agent aromatisant et colorant alimentaire. La curcumine a une longue histoire d'utilisation médicinale pour une grande variété d'affections médicales. L'Organisation mondiale de la santé a approuvé son utilisation en tant qu'additif alimentaire non toxique et médicament potentiellement sûr. Son administration orale est bien tolérée, mais sa biodisponibilité est très faible. Moins de 1 % de la curcumine atteint la circulation sanguine, où le foie détruit rapidement la majeure partie de celui-ci. Un système de véhicule protéique adapté lié de façon covalente à la curcumine avec des groupes chimiques fonctionnels de curcumine naturelle conservés, aisément soluble dans l'eau, aisément digestible, librement absorbé dans l'intestin, de longue demi-vie sérique devrait contribuer à tirer les plus grands effets bénéfiques possibles de ce traitement historique, mais prometteur pour plusieurs maladies aiguës et chroniques. De plus, le conjugué protéine-curcumine pourrait être utilisé en tant qu'additif diététique et colorant alimentaire naturel. En conséquence, sur la base de l'application réussie de notre « brevet en instance PCT/EG2008/000044 » précédent, déposé le 20/11/2008 sous le titre « Long Acting Conserved Natural Functional Groups Curcumin », la nécessité du présent développement paraissait urgente par l'utilisation de plusieurs autres systèmes de véhicule protéique pour la curcumine. L'application clinique réussie d'un système de véhicule protéique globulaire pour la curcumine a été internationalement publiée dans le Journal of International Society for Sexual Medicine et est disponible en ligne depuis le 19 octobre 2009 12h02, sous le titre « Novel Water-soluble Curcumin Derivative Mediating Erectile Signaling ». \|  \| \| **Designated States:** \| AE, AG, AL, AM, AO, AT, AU, AZ, BA, BB, BG, BH, BR, BW, BY, BZ, CA, CH, CL, CN, CO, CR, CU, CZ, DE, DK, DM, DO, DZ, EC, EE, EG, ES, FI, GB, GD, GE, GH, GM, GT, HN, HR, HU, ID, IL, IN, IS, JP, KE, KG, KM, KN, KP, KR, KZ, LA, LC, LK, LR, LS, LT, LU, LY, MA, MD, ME, MG, MK, MN, MW, MX, MY, MZ, NA, NG, NI, NO, NZ, OM, PE, PG, PH, PL, PT, RO, RS, RU, SC, SD, SE, SG, SK, SL, SM, ST, SV, SY, TH, TJ, TM, TN, TR, TT, TZ, UA, UG, US, UZ, VC, VN, ZA, ZM, ZW. African Regional Intellectual Property Org. (ARIPO) (BW, GH, GM, KE, LS, MW, MZ, NA, SD, SL, SZ, TZ, UG, ZM, ZW) Eurasian Patent Organization (EAPO) (AM, AZ, BY, KG, KZ, MD, RU, TJ, TM) European Patent Office (EPO) (AT, BE, BG, CH, CY, CZ, DE, DK, EE, ES, FI, FR, GB, GR, HR, HU, IE, IS, IT, LT, LU, LV, MC, MK, MT, NL, NO, PL, PT, RO, SE, SI, SK, SM, TR) African Intellectual Property Organization (OAPI) (BF, BJ, CF, CG, CI, CM, GA, GN, GQ, GW, ML, MR, NE, SN, TD, TG). \|  \|  \| **Publication Language:** \| English (EN) \| \| --- \| --- \| \| **Filing Language:** \| English (EN) \|  \| \| **Note:** Text based on automatic Optical Character Recognition processes. Please use the PDF version for legal matters \|  \| [Machine translation](http://patentscope.wipo.int/search/en/detail.jsf;jsessionid=8633ED619BCF398226A5E614CF101D5A.wapp1?docId=WO2011100984&recNum=72&maxRec=8021524&office=&prevFilter=&sortOption=Pub+Date+Desc&queryString=&tab=PCTDescription) \| \| --- \| --- \| --- \| \| \| --- \| --- \| --- \| --- \| \| GELATIN , A CURCUMIN DRUG CARRIER SYSTEM  Technical Field  This invention relates to the synthesis of a novel curcumin-protein conjugate as drug carrier system conserving the natural functional groups necessary for curcumin biochemical, physiological and pharmacological functionalities originally possessed by natural curcumin. The novel compound will be submitted to testing in a wide range of variable clinical, pharmaceutical, health food products and as a general natural food colorant.  The invention also relates to a novel intermediate essential for the synthesis of the abovementioned curcumin-protein conjugate, that bears and serves, at the same time, most of the abovementioned functionalities of the novel product, except the presence of the protein carrier property with all of the other properties related to it, such as the slow intestinal absorption with all its relevant consequences. The use of protein carriers for several other drugs has been proved to be highly valuable tools as drug delivery systems that carry some specific drugs to sites within the body not reachable or difficult to reach with the proper therapeutic concentration by the drug by itself.  The novel derivatives relate to conveying lacking essential properties on natural curcumin such as; free water solubility, easy digestibility, free intestinal absorption, higher and longer serum half-life and improved original biochemical, physiological and pharmacological potencies of natural curcumin towards certain pre-studied therapeutic effects in humans arid experimental animal models. In addition, the novel derivatives are intended to be used as natural health food additives and as food colorants.  Such novel protein curcumin carrier derivatives with their improved properties are intended to be submitted to testing in medical studies in experimental animal models to try to get better medicinal effects that were not achievable with natural curcumin. Highly controlled clinical trails on volunteer consented humans in internationally accredited clinical research centers will follow based on the terms and rules internationally approved for such purposes.  Background Art  Curcumin, l,7-Bis(4-hydroxy-3-methoxyphenyI)-l,6-heptadiene-3,5-dione is a is water insoluble component of turmeric, a yellow spice from the rhizomes of the herb Curcuma longa L. (Zingiberaceae), that is widely used as a food flavoring and coloring agent. Curcumin has a long history of medicinal use for the treatment of a wide variety of medical conditions; The World Health Organization approved its use as non-toxic food additive and a potentially safe drug.  Oral administration of curcumin is well tolerated, but its bioavailability is very low. However, when curcumin is used as a supplement, it is absorbed quickly but does not stay in the body for long. The cell lining of the intestine are equipped with enzymes that convert curcumin into other substances, and also equipped with molecular pumps that pump curcumin and its byproducts out of the intestinal lining back into the intestine. Less than 1% of the urcumin actually makes its way to the bloodstream, where the liver rapidly destroys most of it.  Mixing of Bioperine with curcumin is one of the most famous solutions to counteract this problem. Bioperine is a blend from black and long pepper, when used as an additive; it enhances the absorption of curcumin from the gastrointestinal tract. But for people who have medical conditions, or who are taking other supplements or drugs, all kinds of risks are elevated as such substances are known to interact with various drugs used to treat several medical conditions.  In general three different approaches were used to improve curcumin bioavailability; namely: additives, microemulsions and chemical derivatives, but none proved to overcome the problem. Additives such as Piperine, (from black pepper), Quercetin (from various plants) and Genistein (from soy) were used to inhibit the natural pumps that expel curcumin out of the intestinal . and/or other cells. Microemulsions, e.g. natural oils such as peppermint oil, different surfactants such as lecithin, monoolein, Tween-20 were used to facilitate intestinal curcumin absorption in larger amounts. Chemical derivatives e.g., tetrahydro curcumin, demethoxy curcumin, tetrahydrodemethoxy curcumin, bisdemethoxy curcumin, tetrahydro bisdemethoxy curcumin, hexahydrocurcumin, acetate, phosphate, gluconate, etc. were used with the aim to try to avoid the abovementioned action of the intestinal enzymes/pumps and liver enzymes. A serious drawback was that all such chemical derivatives were made through the use of the curcumin natural functional chemical groups required for full biological activity, (biochemical, physiological and pharmacological potencies).  On the other hand, no protein carriers for curcumin, based on covalent conjugation of curcumin to a carrier protein were previously reported. Some other reported systems made use of the natural curcumin-protein interactions. Obviously such curcumin carrier protein interactions are subject to several dissociating factors, starting and not ending with the gastrointestinal tract.  A suitable protein carrier system covalently linked to curcumin with conserved natural curcumin functional chemical groups, of free water solubility, easy digestibility, free intestinal absorption, long serum half-life should help getting the utmost possible beneficial effects of this historical, albeit promising treatment for several acute and chronic illnesses. Additionally, the protein-curcumun conjugate could be used as health food additive and a natural food colorant.  Accordingly, based on one of the successful applications of our previous "Patent Pending PCT/EG2008/000044" filed on 20/11/2008 by the same first author; REZQ, El-Sayed Ameen Mahmoud et al., under "Long Acting Conserved Natural Functional Groups Curcumin", the need looked urgent to develop several other protein carrier systems for curcumin. The successful clinical application of a globular carrier protein system for curcumin has been internationally published in the Journal of International Society for Sexual Medicine and is available online since Oct. 19 2009 12:02PM, under "Novel Water-soluble Curcumin Derivative Mediating Erectile Signaling". Highly trustful of the originality of the scientific research hypothesis and the results outcome, the "Journal Editorial Board" didn't ask for the disclosure of the invention.  The same "Patent Pending PCT/EG2008/000044" has also been granted a national project fund of L.E. 1,000,000 From the Academy of Scientific Research and Technology of Egypt, once again, without asking for the disclosure of the invention. The project entitled "Development of a Novel Drug of Herbal Origin for Management of Erectile Dysfunction and Diabetes Mellitus" under ID: 634, the Science and Technology Fund (STDF).  Disclosure of Invention  Curcumin, l,7-bis(4-hydroxy-3-methoxyphenyl)-l,6-heptadiene-3,5-dione (I), was coupled to diazotized 4-aminobenzoic acid for the synthesis of the novel compound l,7-bis(5-carboxyphenylazo-4-hydroxy-3-methoxyphenyl)-l,6-heptadiene-3,5-dione (II), which in turn was utilized for the synthesis of the novel curcumin-gelatin as a glutinous conjugate (III), through the use of l-ethyl-3-(3-dimethylaminopropyl) carbodiimide hydrochloride, EDC. Both compounds (II) and (III) represent the novel embodiment of this invention.  Nitrous acid was generated by the addition of a solution of 0.85 mEq of sodium nitrite to an excess of IN HC1 with continuous stirring in ice bath. The reaction was maintained at a temperature of 5°C. A solution of 0.85 mEq of 4-aminobenzoic acid in IN HC1 chilled to 5°C was prepared with continuous stirring in ice bath for 20 minutes; the pH of 1.0 was never exceeded. The 4-aminobenzoic acid solution was then added slowly to the cold freshly prepared nitrous acid with continuous stirring in ice bath at5°C. Diazotized 4-minobenzoic acid was added dropwise to an equivalent concentration, (0.85 mEq) of curcumin, (I) dissolved in ethanol/lN NaOH at pH 11.0 with continuous stirring at 5°C. The solution was acidified with IN HC1 to pH 2.0 at which derivative (II) l,7-bis(5-carboxyphenylazo-4-hydroxy-3-methoxyphenyl)-l,6-heptadiene-3,5-dione was precipitated. The precipitate was centrifuged and redissolved in ethanol/lN NaOH at pH 11.0 again. After repeating the acid and base cycle twice, the crude derivative (II) was chromatographed on a column of silica gel. Reduced pressure and temperature evaporation of the elution solvent gave a derivative of about 98% purity as checked by TLC.  The curcumin-gelatin conjugate (III), of this invention was synthesized in a medium of 1% NaCl/l,4-dioxane/lN NaOH solution of pH 8-10, at 5°C with continuous stirring, by adding previously cooled to 5°C, 0.1 solution of l-ethyl-3-(3-dimethylaminopropyl) carbodiimide hydrochloride, EDC to the equivalent concentration of purified crystalline l,7-bis(5-carboxyphenylazo-4-hydroxy-3-methoxyphenyl)-l,6-heptadiene-3,5-dione, (II) in the same medium with continuous stirring. One per cent gelatin solution in 0.5N NaOH was added to the foregoing mixture at 5°C, pH 8-10 with continuous stirring for 1 hr until the intermediate azopseudourea has been completely conjugated to gelatin as was evident by the  complete disappearance of the original red color of the l,7-bis(5-carboxyphenylazo-4-hydroxy-3-methoxyphenyl)-l,6-heptadiene-3,5-dione, (II) solution, after which the mixture was centrifuged off, acidified to pH 5.1, salted out with either solid NaCl or ammonium sulphate, recentrifuged, redissolved then dialyzed for 24 hr at 5°C against 0.5M sodium carbonate, pH 8.2 until any color no longer appears in dialysis solution. A final dialysis is performed against bi-distilled water for 24 hours at 5°C, after which the protein conjugate (III) was lyophilized. The novel curcumin derivatives (II) and (III) were characterized by IR Spectroscopy, GC/MS, NMR, EM, TLC, Gel Filtration, Continuous Flow Electrophoresis and Isoelectric Focusing whenever applicable. \|  \| \| **Note:** Text based on automatic Optical Character Recognition processes. Please use the PDF version for legal matters \|  \| [Machine translation](http://patentscope.wipo.int/search/en/detail.jsf?docId=WO2011100984&recNum=72&tab=PCTClaims&maxRec=8021524&office=&prevFilter=&sortOption=Pub+Date+Desc&queryString=) \| \| --- \| --- \| --- \| \| \| --- \| --- \| --- \| --- \| \| Claims  We claim the following:  1- The synthesis of a novel covalently linked drug carrier system of gelatin-curcumin conjugate with conserved natural functional chemical groups of curcumin, of free water solubility, easy digestibility, and free intestinal absorption.  2- According to claim (1) we claim higher curcumin bioavailability and longer serum half-life for on oral administration of the novel gelatin-curcumin conjugate.  3- According to claims (1&2) we claim the novel derivative to convey lacking essential properties on natural curcumin such as; free water solubility, easy digestibility, free intestinal absorption, higher and longer serum half-life and improved original biochemical, physiological and pharmacological potencies of natural curcumin towards certain pre-studied therapeutic effects in humans and experimental animal models.  4- According to claims (1,2&3) the novel gelatin-curcumin conjugate is intended to be used as therapeutic protein-curcumin carrier system, as a natural health food additives and as a food colorant.  5- We also claim the synthesis of a novel intermediate essential for the synthesis of the novel gelatin-curcumin conjugate.  6- According to claim (4&5) we claim the use of the claimed curcumin derivatives in medical studies in experimental animals for treatment from some acute and chronic illnesses e.g. erectile dysfunction, diabetes mellitus, osteoporosis, cancer and all other illnesses reported to be treated or benefited from natural curcumin treatment.  The novel gelatin curcumin carrier system derivatives with their improved properties are intended to be submitted to testing in medical studies in experimental animal models to try to get better medicinal effects that were not achievable with natural curcumin.  7- According to claims (1-6) we claim the successful conversion of natural pure water insoluble curcumin to a novel, water soluble conserved natural functional group derivatives, with no change to its original active functional natural molecular chemical groups necessary for full biochemical, physiological and pharmacological potencies previously reported for natural curcumin. The novel curcumin derivatives were characterized by IR Spectroscopy, GC/MS, NMR, EM, TLC, Gel Filtration, Continuous Flow Electrophoresis and Isoelectric Focusing whenever applicable. \| \| \| --- \| --- \| --- \| --- \| --- \| --- \| --- \| --- \| --- \| --- \| --- \| --- \| --- \| --- \| --- \| --- \| --- \| --- \| --- \| --- \| --- \| --- \| --- \| --- \| --- \| --- \| --- \| --- \| --- \| --- \| --- \| --- \| --- \| --- \| --- \| --- \| --- \| --- \| --- \| --- \| --- \| --- \| --- \| --- \| --- \| --- \| --- \| --- \| --- \| --- \| --- \| --- \| --- \| --- \| --- \| --- \| --- \| --- \| --- \| --- \| |

Bottom of Form
